# Heavy Resistance Training Versus Plyometric Training for Improving Running Economy and Running Time Trial Performance: A Systematic Review and Meta-analysis

**DOI:** 10.1186/s40798-022-00511-1

**Published:** 2022-11-12

**Authors:** Yuuri Eihara, Kenji Takao, Takashi Sugiyama, Sumiaki Maeo, Masafumi Terada, Hiroaki Kanehisa, Tadao Isaka

**Affiliations:** Faculty of Sport and Health Science, Ritsumeikan University, Shiga, Japan

**Keywords:** Concurrent training, Running performance, Long-distance runners

## Abstract

**Background:**

As an adjunct to running training, heavy resistance and plyometric training have recently drawn attention as potential training modalities that improve running economy and running time trial performance. However, the comparative effectiveness is unknown. The present systematic review and meta-analysis aimed to determine if there are different effects of heavy resistance training versus plyometric training as an adjunct to running training on running economy and running time trial performance in long-distance runners.

**Methods:**

Electronic databases of PubMed, Web of Science, and SPORTDiscus were searched. Twenty-two studies completely satisfied the selection criteria. Data on running economy and running time trial performance were extracted for the meta-analysis. Subgroup analyses were performed with selected potential moderators.

**Results:**

The pooled effect size for running economy in heavy resistance training was greater (*g* = − 0.32 [95% confidence intervals [CIs] − 0.55 to − 0.10]: effect size = small) than that in plyometric training (*g* = -0.13 [95% CIs − 0.47 to 0.21]: trivial). The effect on running time trial performance was also larger in heavy resistance training (*g* = − 0.24 [95% CIs − 1.04 to − 0.55]: small) than that in plyometric training (*g* = − 0.17 [95% CIs − 0.27 to − 0.06]: trivial). Heavy resistance training with nearly maximal loads (≥ 90% of 1 repetition maximum [1RM], *g* = − 0.31 [95% CIs − 0.61 to − 0.02]: small) provided greater effects than those with lower loads (< 90% 1RM, *g* = − 0.17 [95% CIs − 1.05 to 0.70]: trivial). Greater effects were evident when training was performed for a longer period in both heavy resistance (10–14 weeks, *g* = − 0.45 [95% CIs − 0.83 to − 0.08]: small vs. 6–8 weeks, *g* = − 0.21 [95% CIs − 0.56 to 0.15]: small) and plyometric training (8–10 weeks, *g* = 0.26 [95% CIs − 0.67 to 0.15]: small vs. 4–6 weeks, *g* = − 0.06 [95% CIs 0.67 to 0.55]: trivial).

**Conclusions:**

Heavy resistance training, especially with nearly maximal loads, may be superior to plyometric training in improving running economy and running time trial performance. In addition, running economy appears to be improved better when training is performed for a longer period in both heavy resistance and plyometric training.

**Key Points**

1. Heavy resistance training as an adjunct to running training would be more effective in improving running economy and running time trial performance than plyometric training.
2. Resistance training with nearly maximal load (≥ 90% of 1RM or ≤ 4RM) would be more effective to improve running economy.
3. Heavy resistance and plyometric training should be conducted over ≥ 10 weeks to better improve running economy.

## Background

For long-distance runners, running time trial performance is influenced by several physiological parameters, such as maximal oxygen uptake ($\dot{V}$O_2max_), running economy, and lactate threshold [1, 2]. Notably, running economy, defined as the oxygen or metabolic cost required to cover a given distance or to maintain a given speed at a submaximal speed [3], is considered to play an important role in running time trial performance [4–6]. Therefore, mounting studies have investigated the identification of an effective training modality that contributes to improvements in running economy and running time trial performance, as an adjunct to daily running training.

Heavy resistance and plyometric training, which are effective to enhance neuromuscular function, have recently drawn researchers’ attention as a potential training modality that improves running economy and running time trial performance [7, 8]. The reason behind this interest is that the energy cost of skeletal muscle represents majority of the total energy cost of running [9, 10]. Heavy resistance training can increase muscular strength and/or power by changing motor unit recruitment patterns and firing frequency during voluntary muscle contractions [11, 12]. An increase in muscle strength could lower the relative intensity of the load for exercising muscles during running [9]. Consequently, it may contribute to improvements in running economy and running time trial performance [9, 13]. Plyometric training, which mainly consists of various jumping actions utilizing the stretch–shortening cycle (SSC) [14], enhances the ability to store and utilize elastic energy more efficiently [14], leading to a decrease in energy consumption during running [15]. Thus, both heavy resistance and plyometric training may be effective training modalities for improving running economy and running time trial performance.

While strength and conditioning specialists can utilize both heavy resistance and plyometric training to enhance running performance, Li et al. [16] suggest that plyometric training would be more beneficial for improving running economy at faster running speeds compared to heavy resistance training. Based on the findings from their study [16], the magnitude of training effects on running economy and running time trial performance may differ between heavy resistance and plyometric training. For many strength and conditioning professionals, the choice of training modalities is critical to improve the athletes’ performance efficiently and effectively in a limited time. However, it is unknown whether heavy resistance or plyometric training, as an adjunct to running training, is more effective than the other in improving running economy and running time trial performance in long-distance runners. Therefore, the purpose of this systematic review and meta-analysis was to compare the magnitude of the effects of heavy resistance and plyometric training, as an adjunct to distance running training, on running economy and running time trial performance in long-distance runners. By providing a quantitative estimate of the magnitude of the effects of heavy resistance and plyometric training, our systematic review and meta-analysis provide a new perspective on the evidence of training strategies to improve running economy and running time trial performance.

## Methods

### Literature Search Strategy

The systematic review and meta-analysis were conducted in accordance with the Preferred Reporting Items for Systematic Reviews and Meta-Analysis (PRISMA) [17]. The first author (YE) performed comprehensive searches for articles in the electronic databases of PubMed, Web of Science, and SPORTDiscus with the following search terms and Boolean operators: ("strength training" OR "plyometric training" OR "explosive training" OR "resistance training" OR "weight training" OR "concurrent training" OR "muscle training" OR "isometric training" OR "concentric training" OR "eccentric training" OR "depth jumps" OR "muscular endurance training") AND (running OR marathon OR "distance running" OR "distance runner*" OR "endurance running" OR "endurance runner*" OR "endurance athlete*") AND ("running performance" OR "running economy" OR "time trial" OR "VO2max" OR "oxygen consumption" OR "oxygen uptake" OR "energy cost" OR "blood lactate" OR speed OR "running speed" OR "lactate threshold" OR "run* time") NOT "review." The articles had to be written in English and published up to May 7, 2022.

### Performance Level of the Runners Examined

In highly trained runners, where running economy has already been highly developed through years of endurance training, it may be difficult to produce further improvements in running economy and/or running time trial performance [18]. The training levels for long-distance runners are indirectly represented as the performance values [19, 20]. Thus, we classified the runners in the experimental group into three groups of level 1 (Lv. 1), level 2 (Lv. 2), and level 3 (Lv. 3) based on the $\dot{V}O_{2\max }$ values reported in each study. In this process, $\dot{V}$O_2max_ was normalized ($\dot{V}$O_2maxNor_) using Eq. 1 [7, 8, 21, 22].

1 $$\dot{V}O_{{2\max \;{\text{Nor}}}} = \frac{{\left( {n_{F} - n_{M} } \right) \times 5}}{{n_{F + M} }} + \dot{V}O_{{2\max {\text{BL}}}}$$

where *n*_*F*_ and *n*_*M*_ are the number of female and male participants, respectively, and $\dot{V}$O_2maxBL_ is the mean $\dot{V}$O_2max_ value at baseline. Based on the level of the $\dot{V}$O_2maxNor_, the runners were classified into one of the three categories: $\dot{V}$O_2maxNor_ ≤ 50.0 mL/kg/min for Lv. 1; $\dot{V}$O_2maxNor_ 50–60 mL/kg/min for Lv. 2; $\dot{V}$O_2maxNor_ ≥ 60 mL/kg/min for Lv. 3. If the authors of the selected articles did not report $\dot{V}$ O_2max_ values, we determined runners’ performance level in accordance with the following classification: competition levels of runners (Lv. 1: recreational or local club; Lv. 2: collegiate or provincial; and Lv. 3: national or international), running training history (Lv. 1: ≤ 2 years; Lv. 2: 2–5 years; and Lv. 3: ≥ 5 years), and training period per session (Lv. 1: ≤ 60 min; Lv. 2: 60–120 min; Lv. 3: ≥ 120 min) [21, 23].

### Selection Criteria

We identified studies that evaluated heavy resistance and/or plyometric training and examined the complete text of studies identified through electronic searches to determine if they met the following selection criteria:

- The studies included middle- or long-distance runners (non-runners were defined as untrained or less than 6 months of running training experience). We also adopted studies that targeted cross-country runners, triathletes, and duathletes as participants because they have similar anthropometric characteristics and $\dot{V}$O_2max_ values to those of distance runners [24, 25].
- The studies examined the efficacy of heavy resistance or plyometric training alone. We excluded the studies in which the authors combined heavy resistance training with plyometric training. Heavy resistance training was defined as an exercise in which the maximal load through the intervention was ≥ 70% of 1 repetition maximum (RM) or its equivalent (≤ 12 RM). A study using isometric contraction training with ≥ 70% maximum voluntary contraction was also included [8]. Plyometric training was defined as an exercise with body weight and/or ≤ 20% of 1RM performed by utilizing the SSC [26].
- The training intervention period lasted for 4 weeks or longer. This criterion was employed because neuromuscular adaptations have been observed over even 4 weeks in non-strength-trained individuals [27–29].
- The authors assessed running economy and/or running time trial performance as an outcome measure. The studies were excluded if running economy was measured at a speed yielding a state of respiratory exchange ratio (RER) ≥ 1.00.
- The volume of running training in an endurance-only group, adopted as a control group, was similar to that of an experimental group.
- The complete study was published in a peer-reviewed journal.
- The studies reported the load, number of repetitions, and training types used in the intervention.
- The studies did not include participants with poor health.
- The studies did not use ergogenic substances as part of the intervention.

### Data Extraction

The first author (YE) independently extracted the characteristics of participants (performance levels, number of participants, sex, and age), training protocol, and outcomes on running economy and running time trial performance using standardized forms. The number of participants and mean and standard deviation (SD) values at pre- and post-intervention in each experimental and control group were extracted to calculate Hedges’ *g* and its standard errors (SEs). When a study did not report these numerical values, we contacted the corresponding author of these studies to collect as much data as possible.

### Assessment of Methodologic Quality, Risk of Bias, and Strength of Recommendation

Study quality was assessed using the Physiotherapy Evidence Database (PEDro) scale, Consolidated Standards of Reporting Trials (CONSORT) checklist, and the Oxford level of evidence. The PEDro scale consists of 11 items for rating the methodological quality of randomized controlled trials (RCTs) [30]. Each satisfied item, except for item 1, contributes one point to the total PEDro score (10 = study possesses excellent internal validity and 0 = study has poor internal validity) [30]. CONSORT has been developed to aid authors in presenting the RCTs in a clean, transparent, and complete manner [31]. The CONSORT is composed of a 38-score 25-item checklist, which relates to the reporting of the trial design, analysis, and interpretation of results. When a study was rated 38, the study had an excellent quality of RCTs. Following the critical appraisal, each study was given a level of evidence in accordance with the Oxford Centre for Evidence-Based Medicine guidelines.

The risk of bias was evaluated in accordance with the Cochrane Collaboration’s tool for assessing the risk of bias in the Cochrane handbook [32]. Given that it was impossible to blind the participants, the item of performance bias was removed. Thus, a tool for assessing the risk of bias was composed of selection (random sequence generation and allocation concealment), detection, attrition, and reporting biases. These items were rated as “low risk” or “high risk,” and rated as “unclear” when a study did not report details. A funnel plot and Egger’s test were also used to determine publication bias when a significant result (p < 0.05) was found. The analysis of a funnel plot was conducted for studies examining the effects of heavy resistance or plyometric training on both running economy and running time trial performance.

The strength of recommendation for the included studies was assessed using the Strength of Recommendation Taxonomy (SORT) [33]. The taxonomy consists of A, B, and C ratings. Grade A represents consistent, good-quality, patient-oriented evidence; grade B represents inconsistent or limited-quality, patient-oriented evidence; and grade C represents consensus, usual practice, opinion, and disease-oriented evidence. All assessments of the study were performed by YE.

### Statistical Analyses

We performed a meta-analysis to evaluate the possible effects of heavy resistance and plyometric training on running economy and running time trial performance. Hedges’ *g* and 95% confidence intervals (CIs) were calculated from the sample size, mean, and SD values in each of the experimental and control groups to estimate the magnitude of changes in outcomes between pre- and post-training [34]. The effect sizes in each group were synthesized in the forest plot with a random-effects model. When the included articles included multiple training groups or assessed running economy at several different velocities or running time trial over several different distances, we combined these effect sizes according to the guidelines of Cochrane’s handbook [35]. Additionally, to estimate the effects of heavy resistance or plyometric training as an adjunct to running training, the effect sizes in each of the experimental and control groups were calculated as weighted average by sample size. If effect sizes were provided in the articles, we re-calculated them for consistency by comparing all the studies included in this review. Unless otherwise noted, all data are reported as the mean of Hedges’ *g* [95% CI]. Hedges’ *g* values (regardless of its sign, negative or positive) were interpreted as trivial ≤ 0.2; small 0.2–0.5; moderate 0.5–1.0; and large ≥ 1.0 [36]. When 95% CIs of Hedges’ *g* crossed zero, we interpreted them as meaning that no definitive changes in the outcome were observed [37]. Importantly, improvements in running economy and running time trial performance stand for reduced oxygen/energy cost and time to run a given distance, respectively. Thus, Hedges’ *g* and the percentage change were expressed as negative values when the variables improved. CIs entirely less than zero indicate a significantly beneficial effect of Hedges’ *g*, while CIs entirely greater than zero represent a significantly deleterious effect of Hedges’ *g* [37].

We examined the statistical heterogeneity using the I^2^ and Cochran’s Q tests. The I^2^ values of 25%, 50%, and 75% represented low, moderate, and high heterogeneity, respectively [38]. The Cochran’s Q test was computed, and p values were obtained by comparing the statistic with a *χ*^2^ distribution with k-1 degrees of freedom, where k is the number of adopted studies. A significant Q statistic (*P* < 0.05) suggests that studies are not likely drawn from a common population [39].

In addition, subgroup analyses were performed to determine whether the following variables influenced the improvement in running economy and running time trial performance: (1) performance levels (Lv. 1, Lv. 2, and Lv. 3); (2) age (heavy resistance training, 21.0–31.5 and 34.1–44.8 years; plyometric training, 24.3–31.0 and 32.5–33.3 years); and (3) intervention period (heavy resistance training, 6–8 and 10–14 weeks; plyometric training, 4–6 and 8–10 weeks). In addition, the studies adopting heavy resistance training were categorized as (4) training modality (isometric and dynamic), (5) training intensity (< 90% of 1RM or > 4RM and ≥ 90% of 1RM or ≤ 4RM). The division of the moderator variables was sorted by the median of the studies. All statistical analyses were performed using RStudio (version 2022.02.0 + 443, Boston MA).

## Results

### Study Selection

**Fig. 1:**
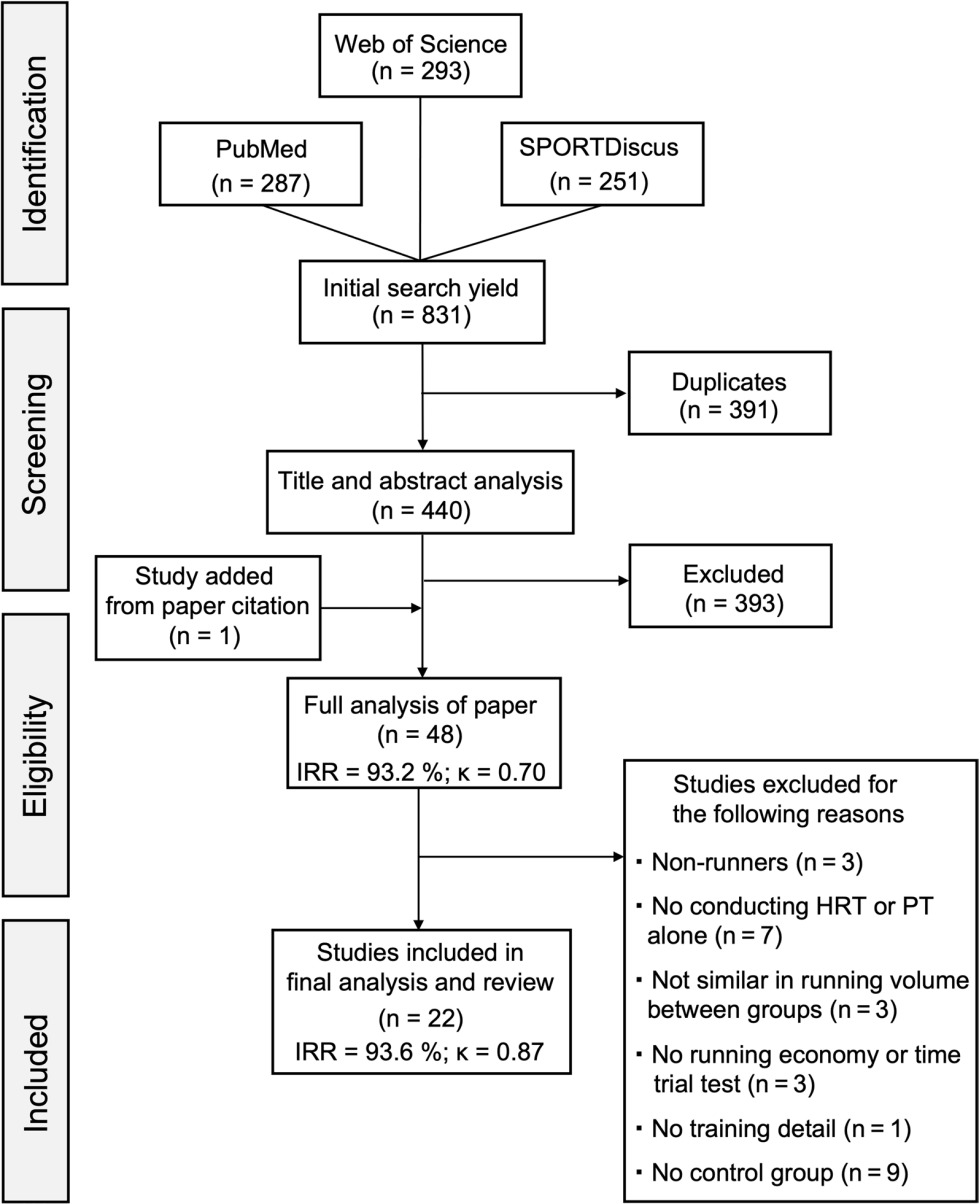
Search, screening, and selection process for suitable studies

Figure 1 provides a visual overview of the selection process in the literature. The study selection process was performed by two independent reviewers (YE and a colleague). Any disagreements were resolved by consensus. The initial search strategy retrieved 831 articles. Following the removal of duplicates (*n* = 391), publications were excluded based on the title and abstract (*n* = 393). One additional record [40] was identified as being potentially relevant via a review article; consequently, 47 studies were considered in detail for appropriateness, resulting in 25 papers [16, 21, 40–62] being excluded from the current review because of insufficient information for data analysis (inter-rater reliability [IRR]: 93.2%, Cohen’s *κ* = 0.70). The reasons for exclusion are shown in Fig. 1. After completion of the exclusion process, 22 articles [63–84] remained (IRR: 93.6%, Cohen’s *κ* = 0.87). Cohen’s κ values were substantial to almost perfect [85]. Among the remaining studies, one research group reported their results across two papers [71, 72]. We considered them as a single study per one research group, and consequently, a total of 21 studies were finally adopted, in which running economy [63–68, 70–75, 78–83] and running time trial performance [65, 67, 69, 76–78, 80–82, 84] were assessed in 18 and 10 studies, respectively. The selected 22 articles were divided into the following training categories: 13 included heavy resistance training [63–75] and 9 included plyometric training [76–84].

### Assessment of Methodologic Quality, Risk of Bias, and Strength of Recommendation

The results of the assessment of the study quality are shown in Table 1. The rating of the study quality as assessed by the PEDro scale was 5.5 ± 0.7. The CONSORT score ranged from 13 to 24, and the mean score was 18.3 ± 2.9. Based on the Oxford evidence level, all studies were appraised as 2b, except for three studies [66, 76, 84] which were rated as 1b.

**Table 1:** Assessment of the study quality and the risk of bias

Study	Quality assessment		The Cochrane Collaboration's tool for assessing the risk of bias					Oxford Evidence levels
	PEDro	CONSORT	Selection bias		Detection bias	Attrition bias	Other bias	
			Random sequence generation	Allocation concealment	Blinding of outcome assessment	Incomplete outcome data	Selective reporting	
Albract and Arampatzis [63]	5	15	High risk	Unclear	Unclear	Low risk	Low risk	2b
Bohm et al. [64]	5	16	Low risk	Unclear	Low risk	High risk	Low risk	2b
Damasceno et al. [65]	6	23	Low risk	Unclear	Low risk	Low risk	Low risk	2b
Ferrauti et al. [66]	6	17	Low risk	Unclear	Low risk	Low risk	Low risk	1b
Festa et al. [67]	5	18	High risk	Unclear	Low risk	High risk	High risk	2b
Karsten et al. [68]	6	18	Low risk	Unclear	Low risk	Low risk	Low risk	2b
Johnston et al. [69]	6	19	Low risk	Unclear	Unclear	Low risk	Low risk	2b
Piacentini et al. [70]	4	24	Low risk	Unclear	Low risk	High risk	Low risk	2b
Vikmoen et al. [71, 72]	4	18	Unclear	Unclear	Low risk	Low risk	Unclear	2b
Fletcher et al. [73]	6	15	Low risk	Unclear	Low risk	Low risk	Low risk	2b
Millet et al. [74]	6	13	Low risk	Unclear	Low risk	Low risk	Low risk	2b
Storen et al. [75]	6	19	Low risk	Unclear	Low risk	Low risk	Low risk	2b
Garcia-Pinillos et al. [76]	6	22	Low risk	Low risk	Low risk	Low risk	Low risk	1b
Machado et al. [77]	6	14	High risk	Unclear	Low risk	Unclear	High risk	2b
Pellegrino et al. [78]	6	19	Low risk	Unclear	Low risk	Low risk	Low risk	1b
Ache-Dias et al. [79]	5	23	Low risk	Unclear	Low risk	Low risk	Low risk	2b
Berryman et al. [80]	5	18	Low risk	Low risk	Low risk	High risk	Low risk	2b
do Carmo et al. [81]	5	21	Unclear	Unclear	Low risk	Low risk	Low risk	2b
Spurrs et al. [82]	6	19	High risk	Unclear	Unclear	Low risk	Low risk	2b
Turner et al. [83]	6	18	Low risk	Unclear	Low risk	Low risk	Low risk	2b
Ramirez-Champillo et al. [84]	6	17	Low risk	Low risk	Low risk	Low risk	Low risk	1b
Mean $\pm$ SD	5.5 ± 0.7	18.4 ± 2.9						

The assessments of PEDro, CONSORT, the risk of bias, and the Oxford Evidence level were graded based on their corresponding checklists

In addition, the mean scores were calculated for PEDro and CONSORT

CONSORT: Consolidated Standards of Reporting Trials; PEDro: Physiotherapy Evidence Database

**Fig. 2:**
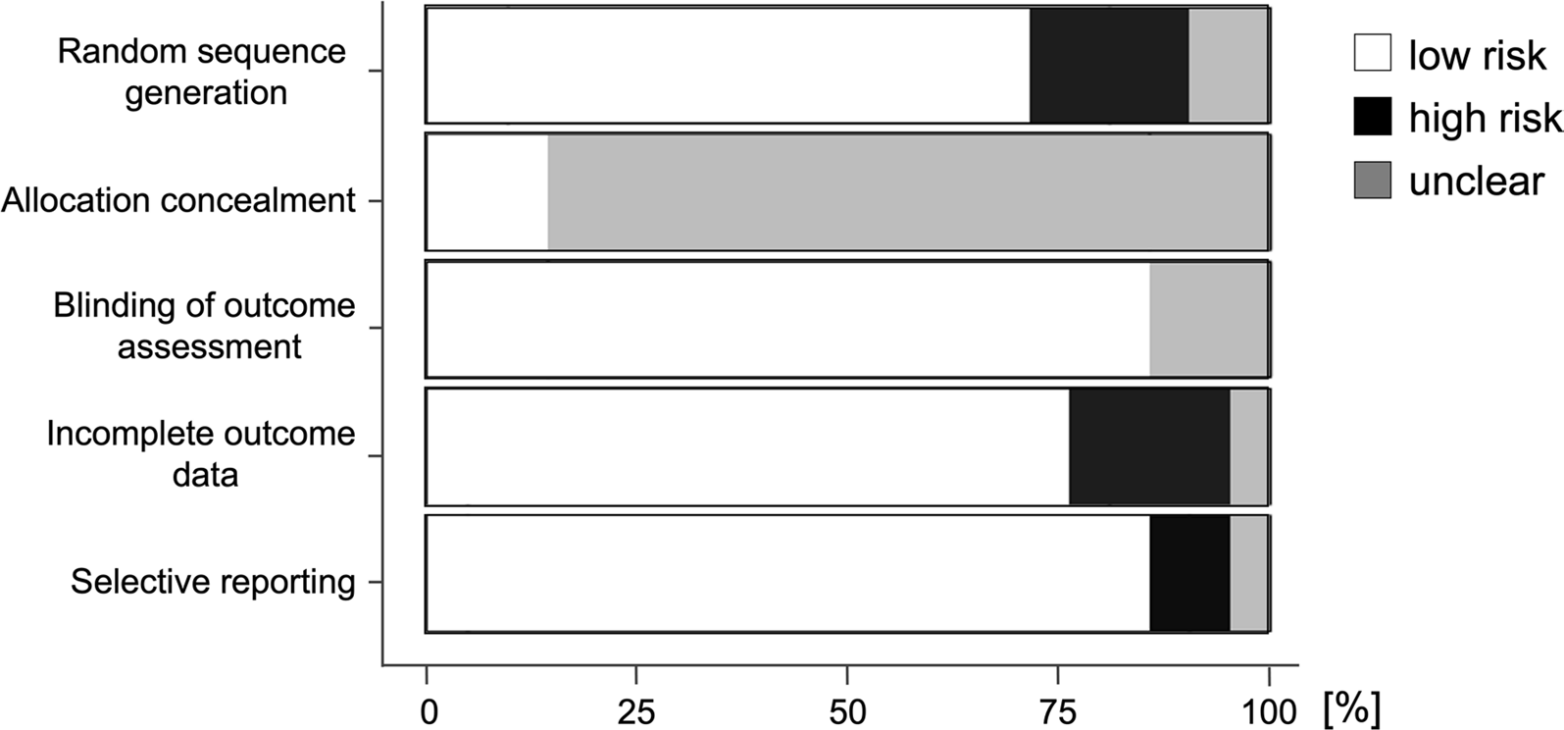
Percentages for the risk of bias

**Fig. 3:**
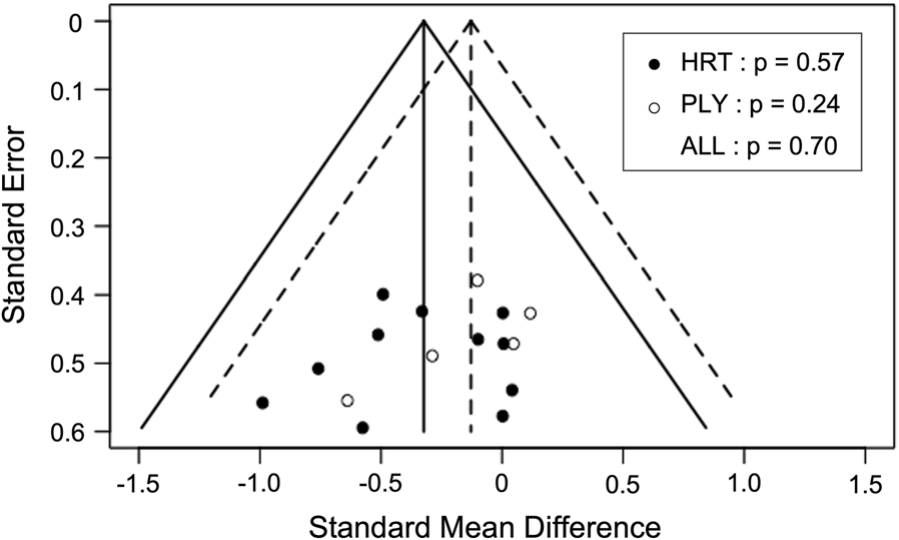
Funnel plots of the studies that examined the effects on running economy. The plot of heavy resistance training (HRT) is shown as solid line; that for plyometric training (PLY) is represented as dash line. Egger’s tests were performed for HRT, PLY, and all plots (ALL)

**Fig. 4:**
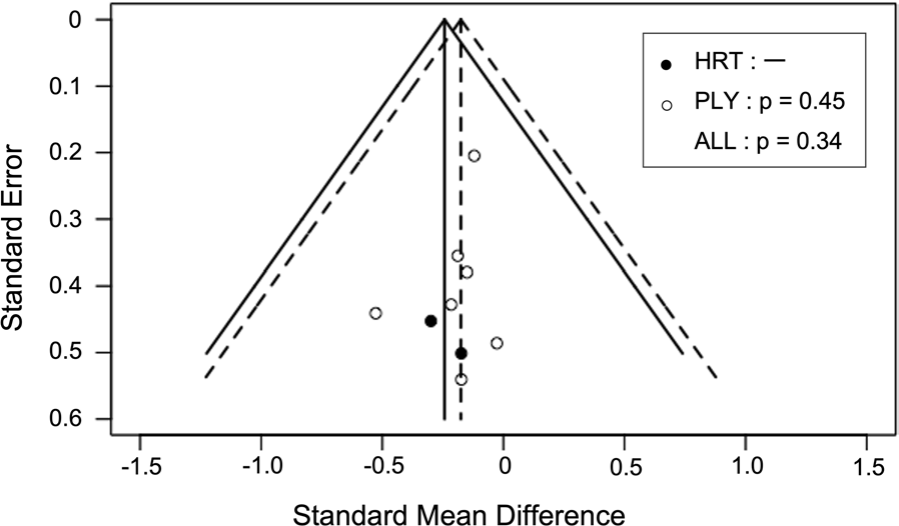
Funnel plots of the studies that examined the effects on running time trial performance. [Legend] The plot of heavy resistance training (HRT) is shown as solid line; that for plyometric training (PLY) is represented as dash line. Egger’s tests were performed for HRT, PLY, and all plots (ALL)

**Fig. 5:**
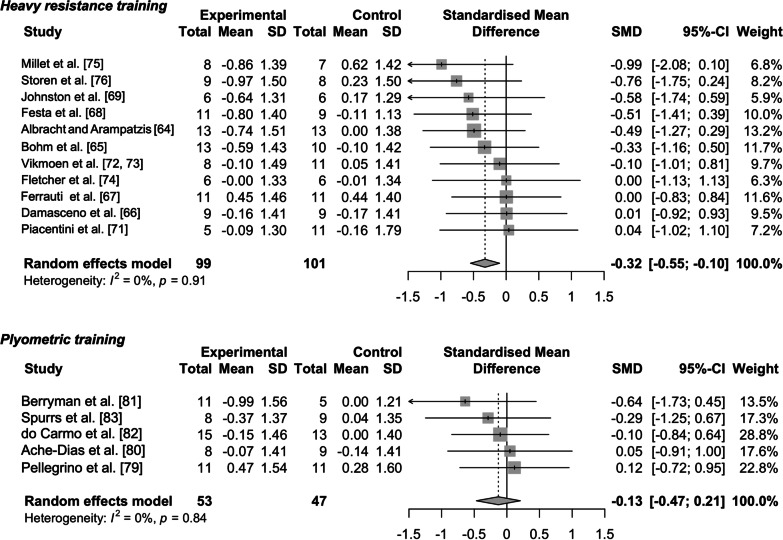
Forest plots of effects of heavy resistance and plyometric training on running economy. Each plot consists of standardized mean difference (SMD) and its 95% CIs. A negative value in SMD represents beneficial effects following heavy resistance or plyometric training as an adjunct to running training, while a positive value in SMD indicates detrimental effects

**Fig. 6:**
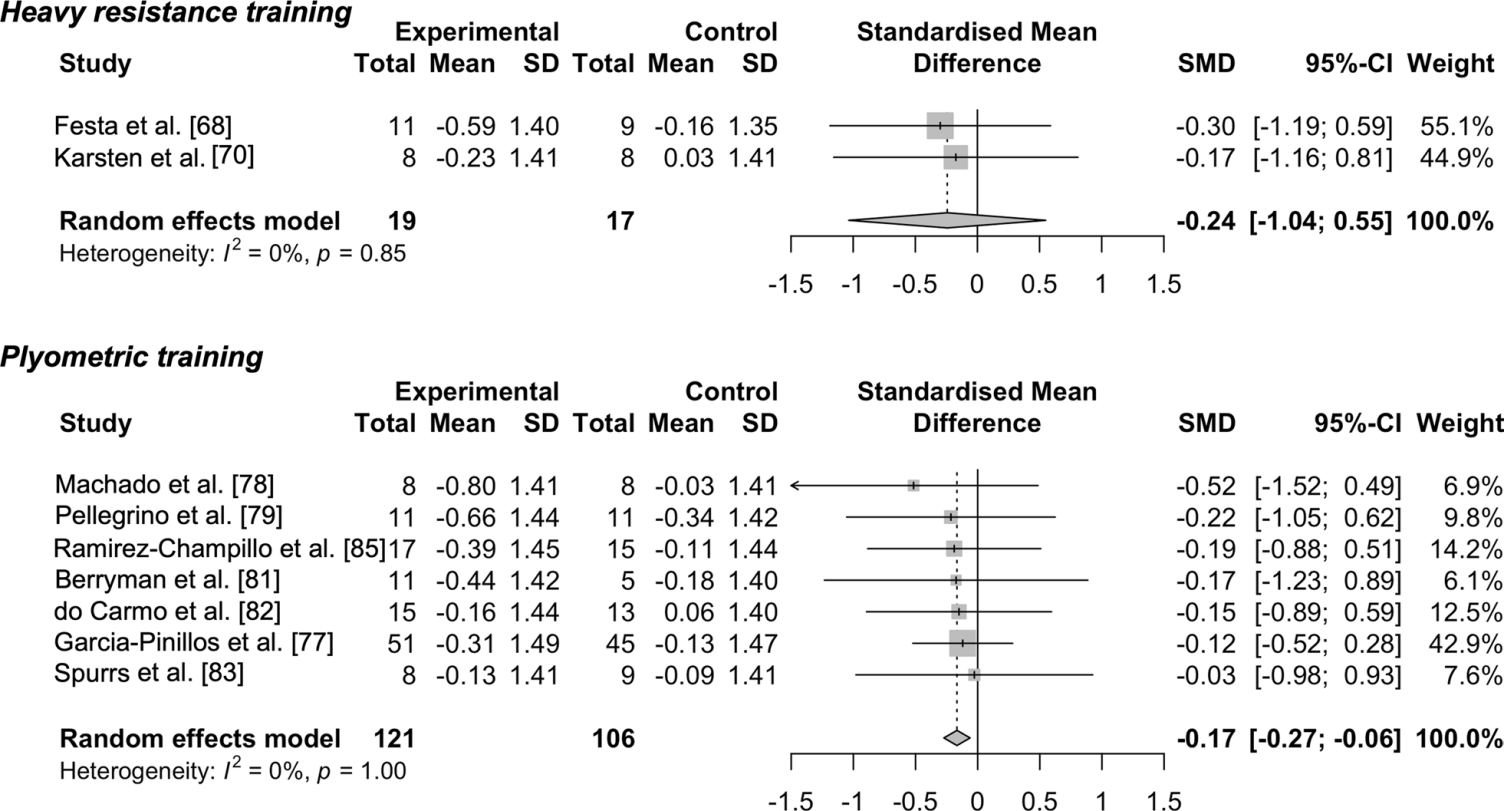
Forest plots of effects of heavy resistance and plyometric training on running time trial performance. Each plot consists of standardized mean difference (SMD) and its 95% CIs. A negative value in SMD represents beneficial effects following heavy resistance or plyometric training as an adjunct to running training, while a positive value in SMD indicates detrimental effects

The results of the assessment of the risk of bias are shown in Table 1 and Fig. 2. With respect to publication bias, all funnel plots indicated a low risk of publication bias (Figs. 3 and 4). I^2^ and Cochran’s Q tests also revealed nonsignificant heterogeneity among the studies examining the effects of heavy resistance and plyometric training on running economy and running time trial performance (Figs. 5 and 6). Additionally, the SORT approach resulted in “B,” a moderate strength of recommendation.

### Effects of Heavy Resistance versus Plyometric Training on Running Economy and Running Time Trial Performance

The numbers of the studies examining the effects of heavy resistance and plyometric training were 14 and 8, respectively, and their total sample sizes were 216 and 263, respectively. Intervention periods tended to differ between heavy resistance and plyometric training (heavy resistance, 9.6 [95% CIs 8.0 to 11.2]; plyometric, 6.9 [95% CIs 5.8 to 8.0]) (Tables 2 and 3).

**Table 2:** Study designs, training variables, and the results of the studies adopting heavy resistance training

Study designs				Training programs			Results	
Performance level	Study	Group	Number of participants: sex, age	Training period (weeks)	Training mode	Maximal intensity through the intervention	Running economy	Running time trial performance
1	Albracht and Arampatzis [63]	HRT	13: M, 27 ± 5	14	IRT	90% MVC	10.8 km/h: $\dot{V}$ O_2_; − 5.0%, *g* = − 0.92 [ − 1.72, − 0.12] ECr; − 4.7%, *g* = − 0.59 [ − 1.37, 0.19] 12.6 km/h:$\dot{V}$ O_2_; − 3.4%, *g* = − 0.55 [ − 1.33 0.23] ECr; − 3.5%, *g* = − 0.46 [ − 1.24 0.32]	–
		Control	13: M, 25 ± 3		–	–	10.8 km/h: $\dot{V}$ O_2_;0.0%, *g* = 0.00 [ − 0.76, 0.76] ECr; 0.0%, *g* = 0.00 [ − 0.76, 0.76] 12.6 km/h: $\dot{V}$ O_2_; 0.0%, *g* = 0.00 [ − 0.76, 0.76] ECr; 0.0%, *g* = 0.03 [ − 0.73, 0.79]	–
1	Bohm et al. [64]	HRT	13: M = 9, F = 4, 29 ± 5	14	IRT	90% MVC	9 km/h: ECr; − 3.8%, *g* = − 0.59 [ − 1.37, 0.19]	–
		Control	10: M = 3, F = 7, 31 ± 3		–	–	9 km/h: ECr; − 0.9% *g* = − 0.10 [ − 1.98, 0.78]	–
1	Damasceno et al. [65]	HRT	9: M, 34.1 ± 7.7	8	DRT	3RM	12 km/h: $\dot{V}$ O_2_; − 1.4%, *g* = − 0.16 [ − 1.08, 0.76]	10 km: − 2.5% (p = 0.039)
		Control	9: M, 32.9 ± 9.2		–	–	12 km/h: $\dot{V}$ O_2_; − 1.9%, *g* = − 0.17 [ − 1.09, 0.75]	10 km: − 0.7% (NS, p ≥ 0.05)
1	Ferrauti et al. [66]	HRT	11: M = 9, F = 2, 40.0 ± 11.4	8	DRT MET	DRT: 3RM MET: 20RM	8.6 km/h: $\dot{V}$ O_2_; 5.1%, *g* = 0.60 [ − 0.26, 1.46] 10.1 km/h: $\dot{V}$ O_2_; 2.2%, *g* = 0.30 [ − 0.54, 1.14]	–
		Control	11: M = 7, F = 4, 40.0 ± 11.4		–	–	8.6 km/h: $\dot{V}$ O_2_; 4.0%, *g* = 0.34 [ − 0.50, 1.18] 10.1 km/h: $\dot{V}$ O_2_; 4.6%, *g* = 0.55 [ − 0.29, 1.39]	–
1	Festa et al. [67]	HRT	11: M = 6, F = 5, 44.2 ± 6.0	8	DRT	No numerical data	8.5 km/h: $\dot{V}$ O_2_; − 6.3%, *g* = − 0.80 [ − 1.63, 0.09]	2 km: − 4.5%, *g* = − 0.47 [ − 1.31, 0.37] 10 km: − 6.1%, *g* = − 0.71 [ − 1.57, 0.15]
		Control	9: M = 6, F = 3, 45.4 ± 8.0		–	–	8.5 km/h: $\dot{V}$ O_2_; 0.8%, *g* = 0.06 [ − 0.85, 0.99]	2 km: − 2.2%, *g* = − 0.18 [ − 1.10, 0.74] 10 km: − 2.5%, *g* = − 0.14 [ − 1.06, 0.78]

Study designs				Training programs			Results	
Performance level	Study	Group	Number of participants: sex, age	Training period (weeks)	Training mode	Maximal intensity through the intervention	Running economy	Running time trial performance
1	Karsten et al. [68]	HRT	8: M = 5, F = 3, 39 ± 5.1	6	DRT	80% 1RM	–	5 km: − 3.5%, *g* = − 0.23 [ − 1.21, 0.75]
		Control	8: M = 6, F = 2, 30 ± 7.7		–	–	–	5 km: 0.5%, *g* = 0.03 [ − 0.95, 1.01]
2	Johnston et al. [69]	HRT	6: F, 30.3 ± 1.4	10	DRT	6RM	12.8 km/h: $\dot{V}$ O_2_; − 4.1%, *g* = − 0.66 [ − 1.82, 0.50] 13.8 km/h: $\dot{V}$ O_2_; − 3.8%, *g* = − 0.61 [ − 1.77, 0.55]	–
		Control	6: F, 30.3 ± 1.4		–	–	12.8 km/h: $\dot{V}$ O_2_; 0.5%, *g* = 0.13 [ − 1.01, 1.27] 13.8 km/h: $\dot{V}$ O_2_; 0.9%, *g* = 0.22 [ − 0.92, 1.36]	–
2	Piacentini et al. [70]	HRT	6: M = 4, F = 2, 44.2 ± 3.9	6	DRT	90% 1RM	9.75 km/h: $\dot{V}$ O_2_; − 0.5%, *g* = − 0.04 [ − 1.18, 1.10] 10.75 km/h: $\dot{V}$ O_2_; − 6.2%, *g* = − 0.62 [ − 1.78, 0.54] 11.75 km/h: $\dot{V}$ O_2_; 2.8%, *g* = 0.24 [ − 0.90, 1.38]	–
		HRT	5: M = 3, F = 2, 44.8 ± 4.4			70% 1RM	9.75 km/h: $\dot{V}$ O_2_; − 1.7%, *g* = − 0.25 [ − 1.50, 1.00] 10.75 km/h: $\dot{V}$ O_2_; − 1.3%, *g* = − 0.19 [ − 1.42, 1.04] 11.75 km/h: $\dot{V}$ O_2_; − 1.2%, *g* = − 0.12 [ − 1.35, 1.11]	–
		Control	5: M, 43.2 ± 7.9		–	–	9.75 km/h: $\dot{V}$ O_2_; 0.0%, *g* = 0.04 [ − 1.19, 1.27] 10.75 km/h: $\dot{V}$ O_2_; − 1.3%, *g* = − 0.19 [ − 1.42, 1.04] 11.75 km/h: $\dot{V}$ O_2_; − 1.2%, *g* = − 0.12 [ − 1.35, 1.11]	–
2	Vikmoen et al. [71, 72]	HRT	11: F, 31.5 ± 8.0	11	DRT	4RM	10 km/h: $\dot{V}$ O_2_; − 0.5%, *g* = − 0.10 [ − 0.98, 0.78]	–
		Control	8: F, 34.9 ± 7.5		–	–	10 km/h: $\dot{V}$ O_2_; 0.3%, *g* = 0.05 [ − 0.93, 1.03]	–

Study designs				Training programs			Results	
Performance level	Study	Group	Number of participants: sex, age	Training period (weeks)	Training mode	Maximal intensity through the intervention	Running economy	Running time trial performance
3	Fletcher et al. [73]	HRT	6: M, 22.2 ± 3.1	8	IRT	80% MVC	12.3 km/h: ECr; 1.0%, *g* = 0.12 [-1.02, 1.26] 13.9 km/h: ECr; − 0.2%, *g* = -0.03 [-1.17, 1.11] 15.6 km/h: ECr; − 0.5%, *g* = -0.10 [-1.24, 1.04]	–
		Control	6: M, 26.3 ± 6.0		–	–	12.3 km/h: ECr; 0.0%, *g* = 0.00 [-1.14, 1.14] 13.9 km/h: ECr; 0.2%, *g* = 0.04 [-1.10, 1.18] 15.6 km/h: ECr; − 0.2%, *g* = -0.06 [-1.20, 1.08]	–
3	Millet et al. [74]	HRT	7: M, 24.3 ± 5.2	14	DRT	90% 1RM	15.0 km/h: $\dot{V}$ O_2_; − 6.9%, *g* = -0.87 [-1.97, 0.23] 17.5 km/h: $\dot{V}$ O_2_; − 5.6%, *g* = -0.85 [-1.95, 0.25]	–
		Control	8: M, 21.4 ± 2.1		–	–	15.0 km/h: $\dot{V}$ O_2_; 7.1%, *g* = 0.74 [-0.28, 1.76] 17.5 km/h: $\dot{V}$ O_2_; 5.4%, *g* = 0.49 [-0.51, 1.49]	–
3	Storen et al. [75]	HRT	8: M = 4, F = 4, 28.6 ± 10.1	8	DRT	4RM	70% of $\dot{V}$ O_2max_: $\dot{V}$ O_2_; − 5.0%, *g* = -0.97 [-2.01, 0.07]	–
		Control	9: M = 5, F = 4, 29.7 ± 7.0		–	–	70% of $\dot{V}$ O_2max_: $\dot{V}$ O_2_; 1.8%, *g* = 0.23 [-0.75, 1.21]	–
Summary		–	Total size 216	9.6 [8.0, 11.2]	–		Weighted average by sample size HRT group: *g* = -0.43 [-0.69, − 0.17] Control group: *g* = 0.07 [-0.06, 0.21]	Weighted average by sample size HRT group: *g* = -0.44 [-0.48, − 0.39] Control group: *g* = -0.07 [-0.20, 0.06]

HRT: heavy resistance training, M: male, F: female, IRT: isometric resistance training, DRT: dynamic resistance training, MET: muscle endurance training, RM: reputation maximum, reps: reputations, wk: week, MVC: maximum voluntary contraction, $\dot{V}$ O_2_: oxygen consumption, ECr: energy cost of running, SD: standard deviation, NS: no significant differences (p ≥ 0.05) between pre and post

*Notation of results:* The results for running economy were represented as “running speed: parameter; percentage changes, Hedges’ *g* [95% CIs lower limit, upper limit]” and running time trial performance, represented as “running distance: percentage changes, Hedges’ *g* [95% CIs lower limit, upper limit].”

Data provided in the paper were described if we could not calculate the effect sizes due to the lack of data

**Table 3:** Study designs, training variables, and the results of the studies adopting plyometric training

Study designs				Training programs		Results	
Performance level	Study	Group	Number of participants: sex, age	Training period (weeks)	Training	Running economy	Running time trial performance
1	Garcia-Pinillos et al. [76]	PLY	51: M = 27, F = 24, 27.2 ± 8.6	10	Jump rope 5 min per 1 session 10–20 min/wk	–	3 km: − 3.0%, *g* = − 0.72 [ − 0.72, 0.10]
		Control	45: M = 24, F = 21, 26.1 ± 6.3		–	–	3 km: − 1.5%, *g* = − 0.13 [ − 0.56, 0.30]
1	Machado et al. [77]	PLY	8: M, 39.0 ± 4.0	8	45-cm drop jump only 6 sets × 30 s with 30 s of recovery	–	10 km: − 11.6%, *g* = − 0.89 [ − 1.91, 0.13]
		Control	8: M, 39.0 ± 4.0		–	–	10 km: − 0.3%, *g* = − 0.03 [ − 1.01, 0.95]
1	Pellegrino et al. [78]	PLY	11: M = 7, F = 4, 32.5 ± 2.0	6	Squat jump, etc 23 sets × 6–15 reps total contacts: 60–228 per session adapted from Spurrs et al. [82]	7.7 km/h: ECr; − 0.5%, *g* = − 0.16 [ − 1.00, 0.68] 9.2 km/h: ECr; − 1.0%, *g* = − 0.38 [ − 1.22, 0.46] 10.6 km/h: ECr; − 1.3%, *g* = − 0.40 [ − 1.24, 0.44] 12.1 km/h: ECr; − 0.8%, *g* = − 0.24 [ − 1.08, 0.60] 13.5 km/h: ECr; 2.3%, *g* = 0.65 [ − 0.21, 1.51] 15.0 km/h: ECr; − 0.3%, *g* = − 0.07 [ − 0.91, 0.77] 16.4 km/h: ECr; 5.8%, *g* = 1.07 [0.17, 1.97]	3 km: − 2.6%, *g* = − 0.66 [ − 1.51, 0.21]
		Control	11: M = 7, F = 4, 34.2 ± 2.6		–	7.7 km/h: ECr; 1.8%, *g* = 0.62 [ − 0.24, 1.48] 9.2 km/h: ECr; 2.3%, *g* = 0.93 [0.05, 1.81] 10.6 km/h: ECr; 2.9%, *g* = 1.01 [0.13, 1.89] 12.1 km/h: ECr; − 0.7%, *g* = − 0.22 [ − 1.06, 0.62] 13.5 km/h: ECr; − 2.8%, *g* = − 0.79 [ − 1.65, 0.07] 15.0 km/h: ECr; − 3.1%, *g* = − 0.81 [ − 1.67, 0.05] 16.4 km/h: ECr; − 4.4%, *g* = − 0.46 [ − 1.30, 0.38]	3 km: − 1.6%, *g* = − 0.34 [ − 1.18, 0.50]
2	Ache − Dias et al. [79]	PLY	9::M = 4, F = 5, 24.3 ± 3.1	4	Continuous jump only 4–6 sets × 30 s with 5 min of recovery	9 km/h: $\dot{V}$ O_2_; − 2.1%, *g* = − 0.14 [ − 1.06, 0.78] ECr; − 2.1%, *g* = − 0.14 [ − 1.06, 0.78]	–
		Control	9::M = 4, F = 5, 31.3 ± 5.7		–	9 km/h: $\dot{V}{O}_{2}$; − 1.3%, *g* = − 0.07 [ − 0.99, 0.85] ECr; − 2.5%, *g* = − 0.14 [ − 1.06, 0.78]	–
2	Berryman et al. [80]	PLY	11: M, 31 ± 7	8	Drop jump (20, 40 or 60 cm) 3–6 sets × 8 reps	12 km/h: $\dot{V}$ O_2_; − 6.9%, *g* = − 0.99 [ − 1.87, − 0.11] $\dot{V}$ O_2_ (kg^−0.75^); − 7.0%, *g* = − 0.94 [ − 1.82, 0.06]	3 km: − 4.8%, *g* = − 0.44 [ − 1.28, 0.40]
		Control	5: M, 29 ± 11		–	12 km/h: $\dot{V}{O}_{2}$; 0.0%, *g* = 0.00 [ − 1.23, 1.23] $\dot{V}$ O_2_ (kg^−0.75^); 0.0%, *g* = 0.00 [ − 1.23, 1.23]	3 km: − 3.0%, *g* = − 0.18 [ − 1.41, 1.05]
2	Do Carmo et al. [81]	PLY	15:M, 33.3 ± 6.1	9	Squat jump, etc 3–5 sets × 6 reps, adapted from Spurrs et al. [82]	average $\dot{V}$ O_2_ of 10 km/h, 12 km/h: − 0.9%, *g* = − 0.15 [ − 0.89, 0.59]	10 km: − 1.0%, *g* = − 0.16 [ − 0.89, 0.57]
		Control	13:M, 33.3 ± 6.1		–	average $\dot{V}$ O_2_ of 10 km/h, 12 km/h: 0.0%, *g* = 0.00 [ − 0.76, 0.76]	10 km: 0.1%, *g* = 0.06 [ − 0.70, 0.82]
2	Spurrs et al. [82]	PLY	8: M, 25 ± 4	6	Squat jump, etc 2–3 sets × 6–15 reps	12 km/h: $\dot{V}$ O_2_; − 6.7%, *g* = − 0.42 [ − 1.42, 0.58] 14 km/h:$\dot{V}$ O_2_; − 6.4%, *g* = − 0.42 [ − 1.42, 0.58] 16 km/h:$\dot{V}$ O_2_ − 4.2%, *g* = − 0.28 [ − 1.26, 0.70]	3 km: − 1.6%, *g* = − 0.13 [ − 1.11, 0.85]
		Control	9: M, 25 ± 4		–	12 km/h:$\dot{V}$ O_2_; 0.5%, *g* = 0.04 [ − 0.88, 0.96] 14 km/h:$\dot{V}$ O_2_; 0.5%, *g* = 0.04 [ − 0.88, 0.96] 16 km/h: $\dot{V}$ O_2_; 0.5%, *g* = 0.04 [ − 0.88, 0.96]	3 km: − 0.5%, *g* = − 0.09 [ − 1.01, 0.80]
2	Turner et al. [83]	PLY	10: M = 4, F = 6, 34 ± 12	6	Vertical jump, etc 5–20 reps per 1 exercise	9.7 km/h: $\dot{V}$ O_2_; NS 11.3 km/h: $\dot{V}$ O_2_; improve, p < 0.05	–
		Control	8: M = 4, F = 4, 27 ± 5		–	9.7 km/h: $\dot{V}$ O_2_; NS 11.3 km/h: $\dot{V}$ O_2_; NS	–
3	Ramirez-Campillo et al. [84]	PLY	17: M = 9, F = 8, 22.1 ± 2.7	6	Drop jump only 2 sets × 10 jumps (20, 40, 60 cm box)	−	2.4 km: − 4.0%, *g* = − 0.39 [ − 1.08, 0.30]
		Control	15: M = 10, F = 5, 22.1 ± 2.7		–	–	2.4 km: − 1.3%, *g* = − 0.11 [ − 0.84, 0.62]
Summary		–	Total size 263	6.9 [5.8, 8.0]	–	Weighted average by sample size PT group: *g* = − 0.21 [ − 0.64, − 0.21] Control group: *g* = 0.05 [ − 0.23, 0.33]	Weighted average by sample size PT group: *g* = − 0.39 [ − 0.54, − 0.24] Control group: *g* = − 0.12 [ − 0.19, − 0.04]

PLY: plyometric training, M: male, F: female, reps: reputations, wk: week, $\dot{V}$ O_2_: oxygen consumption, ECr: energy cost of running, vLT: velocity of lactate threshold,$\dot{V}$ O_2_ (kg^−0.75^): allometric scaling (mL/min/kg^−0.75^), NS: no significant differences (p ≥ 0.05) between pre and post

*Notation of results:* The results of running economy were represented as “running speed: parameter; percentage changes, Hedges’ *g* [95% CI]” and running time trial performance, represented as “running distance, percentage changes, Hedges’ *g* [95% CI].”

Data provided in the paper were described if we could not calculate the effect sizes due to the lack of data

The pooled effect size for heavy resistance training was greater than that for plyometric training (*g* = − 0.32 [small] vs. − 0.17 [trivial]), with the 95% CIs of the former (but not the latter) not crossing zero (Fig. 5). The effect on running time trial performance was also larger in heavy resistance training compared to plyometric training (*g* = − 0.24 [small] vs. − 0.17 [trivial]) although the associated 95% CIs of heavy resistance training crossed zero (Fig. 6).

### Subgroup Analysis on the Effects of Heavy Resistance Training on Running Economy and Running Time Trial Performance

The effect size of heavy resistance training in Lv. 3 runners on running economy was greater than that of Lv. 2 and Lv. 1 runners (Lv. 3 vs. Lv. 1 to 2, *g* = − 0.61 [moderate] vs. − 0.18 to − 0.27 [trivial to small], Table 4). The subgroup difference was also seen in age (young vs. old, *g* = − 0.51 [moderate] vs. − 0.12 [trivial]), training load (≥ 90% of 1RM or ≤ 4RM vs. < 90% of 1RM or > 4RM, *g* = − 0.31 [small] vs. − 0.17 [trivial]), and intervention period (10–14 vs. 6–8 weeks, *g* = -0.45 [small] vs. − 0.21 [small]) (Table 4). The associated 95% CIs of heavier load and longer intervention period did not cross zero. Regarding the running time trial performance, subgroup analyses could not be performed because of the small number of studies examining the effect of heavy resistance training on running time trial performance.

**Table 4:** Subgroup analyses regarding effects of heavy resistance training on running economy

Moderator variables	Hedges’ *g* [95% CIs LL, UL]	Interpretation
*Performance level*		
Lv. 1 (Recreational level)	− 0.27[ − 0.59, 0.04]	Small
Lv. 2 (Moderate level)	− 0.18 [ − 0.92, 0.56]	Trivial
Lv. 3 (High level)	− 0.61 [ − 1.84, 0.63]	Moderate
*Age*		
Young (21.0–31.5 years)	− 0.51 [ − 0.83, 0.19]	Moderate
Old (34.1–44.8 years)	− 0.12 [ − 0.41, 0.17]	Trivial
Training intensity		
< 90% 1RM or > 4RM	− 0.17 [ − 1.05, 0.70]	Trivial
≥ 90% 1RM or ≤ 4 RM	− 0.31 [ − 0.61, − 0.02]	Small
*Training modality*		
Dynamic training	− 0.32 [ − 0.64, 0.00]	Small
Isometric training	− 0.33 [ − 0.89, 0.22]	Small
*Intervention period*		
Short (6–8 weeks)	− 0.21 [ − 0.56, 0.15]	Small
Long (10–14 weeks)	− 0.45 [ − 0.83, − 0.08]	Small

Data are standardized mean difference for effect size values (Hedges’ *g*)

Hedges’ *g* represents time (pre vs. post) by group (experimental vs. control) interaction

CIs: confidence intervals, LL: lower limit, UL: upper limit

### Subgroup Analysis on the Effects of Plyometric Training on Running Economy and Running Time Trial Performance

The effect size of plyometric training in Lv. 2 runners on running economy was greater than that of Lv. 1 runners (Lv. 2 vs. Lv. 1, *g* = − 0.20 [small] vs. 0.12 [trivial]). In addition, the effect size in young runners was larger than that in old runners (young vs. old, *g* = − 0.26 [small] vs. − 0.01 [trivial]), and a long intervention period had a greater effect compared to a short intervention period (8–10 vs. 4–6 weeks, *g* = − 0.26 [small] vs. − 0.06 [trivial]) (Table 5). However, the associated 95% CIs in all subgroups crossed zero. As with heavy resistance training, subgroup analyses could not be performed because of the lack of studies examining the effect of plyometric training on running time trial performance.

**Table 5:** Subgroup analyses regarding effects of plyometric training on running economy

Moderator variables	Hedges’ *g* [95% CIs LL, UL]	Interpretation
*Performance level*		
Lv. 1 (Recreational level)	0.12 [ − 0.72, 0.95]	Trivial
Lv. 2 (Moderate level)	− 0.20 [ − 0.62, 0.22]	Small
Lv. 3 (High level)	−	
*Age*		
Young (24.3–31.0 years)	− 0.26 [ − 1.09, 0.58]	Small
Old (32.5–33.3 years)	− 0.01 [ − 1.38, 1.37]	Trivial
*Intervention period*		
Short (4–6 weeks)	− 0.06 [ − 0.67, 0.55]	Trivial
Long (8–10 weeks)	− 0.26 [ − 0.67, 0.15]	Small

Data are standardized mean difference for effect size values (Hedges’ *g*)

Hedges’ *g* represents time (pre vs. post) by group (experimental vs. control) interaction

CIs: confidence intervals, LL: lower limit, UL: upper limit

## Discussion

The main findings from the current review were that (1) heavy resistance training provided greater effects on both running economy and running time trial performance than plyometric training, (2) subgroup analyses revealed greater effects of heavy resistance training with nearly maximal loads compared with lower loads, and (3) effects on running economy were greater when training was performed for a longer period in both heavy resistance and plyometric training. These results suggest that heavy resistance training, particularly with nearly maximal loads, as an adjunct to running training may be more effective than plyometric training in improving running economy and time trial performance, and both training should be performed for a minimal period (e.g., ≥ 10 weeks) to gain its benefits.

### Effects of Heavy Resistance vs. Plyometric Training on Running Economy and Running Time Trial Performance

Our meta-analysis revealed that heavy resistance training had more beneficial effects on running economy from the perspectives of both the magnitude of the effect size and the associated CIs (Fig. 5). One possible reason for the smaller effects of plyometric training on running economy is the differences in training period between heavy resistance and plyometric training. The average 95% CI of training period in heavy resistance training was 9.6 [95% CIs 8.0 to 11.2] weeks, while that for plyometric training was 6.9 [95% CIs 5.8 to 8.0] weeks. The training period was found to influence the effect on running economy as discussed later, and it has been suggested that plyometric training over ≥ 10 weeks would maximize one’s probability of obtaining significant improvements in jumping performance [86], which could consequently enhance running performance [87, 88]. Despite these previous findings, six of eight studies have conducted plyometric training for 6 weeks or shorter [78, 79, 82–84], which may not have been sufficient to substantially improve running economy. While there is room for future consideration [89], we may say that plyometric training over ≥ 10 weeks period would be needed to improve running economy.

While the effect sizes of heavy resistance training on running time trial performance were greater than those of plyometric training, the CIs around the effect sizes for heavy resistance training crossed zero. The reason for this might be the limited number of studies examining the effects of heavy resistance training on running time trial performance. For example, Damasceno et al. [64] found that heavy resistance training significantly improved 10-km time trial performance although their study was not included in the current meta-analysis since the numerical data were not reported. Thus, a greater number of studies investigating the effects of heavy resistance training on running time trial performance would more clearly show the beneficial effects.

Overall, although heavy resistance training provided greater effects on both running economy and running performance when compared to plyometric training, the effect sizes were small even for heavy resistance training. Thus, long-distance runners and their coaches should not overestimate the effects of both training modalities. Indeed, running economy and running performance have been shown to be underpinned by numerous variables including but not limited to running biomechanics other than neuromuscular functions [90, 91]. Nevertheless, we have also found that runners’ physiological characteristics and training variables influence the effects of both heavy resistance and plyometric training. Hereafter, we discuss each of such potential moderators for a better understanding of the effects of both training modalities.

### Subgroup Analysis on the Effects of Heavy Resistance Training on Running Economy

We additionally conducted subgroup analyses regarding the effects of heavy resistance training on running economy although this analysis on running time trial performance was not performed due to the lack of a number of studies. As a result, heavy resistance training provided significant beneficial effects on running economy when the training period, the age of the runners, and training intensity were treated as moderators. First, training intervention over ≥ 10 weeks had a greater positive effect on running economy (*g* = − 0.45 [95% CIs − 0.83 to − 0.08]) compared with shorter training period. This agrees with previous findings that have identified clear beneficial effects following 12–14 weeks of heavy resistance training [7]. Although short-term such as 4-week heavy resistance training could increase muscle strength [27–29], further gains in muscle strength can be achieved over 8–12 weeks of intervention [92]. The suggestion that the continuation of training over long periods is needed to further enhance running economy may be especially true for highly trained runners. For example, Fletcher et al. [73], who examined the effectiveness of an 8-week heavy resistance training program in international runners, did not observe an improvement in running economy. However, Miller et al. [74] reported that a 14-week heavy resistance training program produced 5.6%–6.9% improvements in running economy among highly trained runners. Considering these findings together with the current results of the subgroup analyses, it is likely that highly trained runners may need to implement heavy resistance training for ≥ 12 weeks to improve running economy.

Furthermore, there were few differences in the effect sizes between the modalities of heavy resistance training (dynamic vs. isometric; *g* = − 0.32 [95% CIs − 0.64 to 0.00] vs. *g* = − 0.33 [95% CIs − 0.89 to 0.22]). The observed similarity would arise from the development of muscle strength induced by these training modalities [93]. An increase in maximal muscle strength of the lower limbs would lower the relative intensity for exercising muscles during running at a given submaximal running speed [9]. Moreover, the biomechanical similarity between dynamic heavy resistance training and running actions would produce a significant positive effect on running economy [94, 95]. On the other hand, isometric heavy resistance training in ankle plantar flexion develops the plantar flexor muscle strength and alters the Achilles tendon properties [63, 64, 73]. The stiffness of the Achilles tendon has been shown to be significantly and negatively related to $\dot{V}$O_2_ during running [96]. Bohm et al. [64] suggested that increased plantar flexor muscle strength and Achilles tendon stiffness, induced by heavy isometric training, reduced the metabolic energy cost associated with contracting the soleus muscle during running, resulting in an improvement in running economy. In any case, considering the slight differences between the effects of dynamic and isometric heavy resistance training on running economy, the current results suggest that both training modalities are useful for improving running economy effectively.

In addition, subgroup analyses also showed that while heavy resistance training in young runners (21.0–31.5 years) produced moderate improvements in running economy (*g* = − 0.51 [95% CIs − 0.83 to − 0.19]), the corresponding gain in middle-aged runners (34.1–44.8 years) was trivial (*g* = − 0.12 [95% CIs − 0.41 to 0.17]). This effect of age on running economy might be influenced by other moderator variables. For example, high-level (Lv. 3) runners were included among young runners (22.2–28.6 years), and they gained moderate beneficial effects on running economy. Furthermore, most previous studies targeting middle-aged runners have set the training period to ≤ 10 weeks. However, Piacentini et al. [70] demonstrated that 85%–90% of 1RM with heavy resistance training yielded a 6.2% running economy improvement in Lv. 2 and master runners, although the intervention period was short (6 weeks). Our subgroup analyses showed that nearly maximal (≥ 90% of 1RM or ≤ 4RM) resistance training provided significant improvements in running economy. Nearly maximal resistance training increases the number of motor units recruited during the maximal voluntary contractions [97, 98] and is responsible for promoting maximal strength adaptation [99]. The increased maximal strength induced by nearly maximal resistance training leads to a lower relative intensity level during running [9]. Thus, there is a possibility that neuromuscular adaptations induced by nearly maximal resistance training might promote improvements in running economy regardless of the runners’ age, competition levels, and training period.

### Subgroup Analysis on the Effects of Plyometric Training on Running Economy

Subgroup analysis clarified that the effects of plyometric training on running economy were smaller for Lv. 1 runners than Lv. 2 runners. This result may be attributed to the low jump ability of Lv. 1 runners due to weak muscle strength. The only study that employed Lv. 1 runners [78] showed that plyometric training provided beneficial effects on running economy at 7.7–10.6 km/h, but detrimental effects on running economy at 12.1–16.4 km/h. Previous studies reported that when the running speed changed from 7.2 to 18.7 km/h, the contact times during the stance period reduced from 343 to 188 ms [100, 101]. It has also been found that the enhancement of jumping ability with short contact times induced by plyometric training played an important role in running economy improvement at a faster speed [16]. Therefore, jumping training with short contact times is required to improve the running economy at fast running speeds. However, individuals with weaker lower extremity strength demonstrated longer ground contact times during drop jumps [102]. Runners with high running performance have been shown to have high muscle strength in the lower extremities [103]. Thus, it seems that the force generation capacity of the lower extremities in Lv. 1 runners would be low and they might perform jumping trainings with longer ground contact times, and consequently, Lv. 1 runners could not improve running economy at a fast running speed, leading to small beneficial effects on the overall running economy.

On the other hand, jump training with short ground contact times could provide similar beneficial effects on running economy as heavy resistance training. The effect size of plyometric training in Lv. 2 runners, who would have greater force generation capacity on running economy, was greater than that in Lv. 1 runners. Moreover, Li et al. [16] found that plyometric training had positive effects on running economy at 16 km/h in Lv. 3 runners, but this result was not found in heavy resistance training. Therefore, paying more attention to the contact times of jumping training would lead to a specific improvement in running economy at fast running speeds.

### Limitations

This study is not without limitations. First, the present study did not consider the effects of running training. This is because some studies described running training volume as the distance, while others reported the duration. However, it is known that conducting heavy resistance training has a beneficial effect on running economy despite the reduction in running training volume [48]. Thus, heavy resistance training as an adjunct to running training may improve running economy, regardless of the running training volume. Nevertheless, future studies should be directed toward investigating the effects of heavy resistance and/or plyometric training as an adjunct to different volumes of running training on running economy and/or running time trial performance.

Second, we calculated the effect size for the magnitude of improvement in running economy and running time trial performance. However, it should be acknowledged that we did not directly compare the magnitude of effect sizes between heavy resistance training and plyometric training because of the difference in training period and the number of studies. Moreover, the lack of information on the effect sizes of heavy resistance and plyometric training on running time trial performance hindered subgroup analyses. Future interventions should directly compare the magnitude of the improvement in running economy and time trial performance between heavy resistance and plyometric training. The findings and limitations of this study will be useful for such future work.

## Conclusions

The present study indicated that as adjunct to running training in long-distance runners, heavy resistance training might be more effective to improve running economy and running time trial performance than plyometric training. Subgroup analysis revealed that nearly maximal (≥ 90% of 1RM or ≤ 4RM) resistance training would lead to greater improvements in running economy, and longer training period resulted in greater effects on running economy in both training modalities. These results indicate that long-distance runners and their coaches may need to consider adopting nearly maximal loads when implementing heavy resistance training, and/or for a long intervention period for both training modalities.

## Data Availability

All data and material reported in this systematic review are from peer-reviewed publications.

## Abbreviations

CI: Confidence interval
CONSORT: Consolidated Standards of Reporting Trials
IRR: Inter-rater reliability
Lv.: Level
PEDro: Physiotherapy Evidence Database
PRISMA: Preferred Reporting Items for Systematic Reviews and Meta-analysis
RCT: Randomized controlled trial
RER: Respiratory exchange ratio
RM: Repetition maximum
SD: Standard deviation
SE: Standard error
SORT: Strength of Recommendation Taxonomy
SSC: Stretch–shortening cycle
$\dot{V}$ O_2max_: Maximum oxygen uptake
$\dot{V}$ O_2maxNor_: Normalized maximum oxygen uptake
